# Robust flux balance analysis of multiscale biochemical reaction networks

**DOI:** 10.1186/1471-2105-14-240

**Published:** 2013-07-30

**Authors:** Yuekai Sun, Ronan MT Fleming, Ines Thiele, Michael A Saunders

**Affiliations:** 1 Institute for Computational and Mathematical Engineering, Stanford University, Stanford, USA; 2 Center for Systems Biology, University of Iceland, Reykjavik, Iceland; 3 Luxembourg Centre for Systems Biomedicine, University of Luxembourg, Campus Belval, Esch-sur-Alzette, Luxembourg; 4 Department of Management Science and Engineering, Stanford University, Stanford, USA

## Abstract

**Background:**

Biological processes such as metabolism, signaling, and macromolecular synthesis can be modeled as large networks of biochemical reactions. Large and comprehensive networks, like integrated networks that represent metabolism and macromolecular synthesis, are inherently multiscale because reaction rates can vary over many orders of magnitude. They require special methods for accurate analysis because naive use of standard optimization systems can produce inaccurate or erroneously infeasible results.

**Results:**

We describe techniques enabling off-the-shelf optimization software to compute accurate solutions to the poorly scaled optimization problems arising from flux balance analysis of multiscale biochemical reaction networks. We implement lifting techniques for flux balance analysis within the openCOBRA toolbox and demonstrate our techniques using the first integrated reconstruction of metabolism and macromolecular synthesis for *E. coli*.

**Conclusion:**

Our techniques enable accurate flux balance analysis of multiscale networks using off-the-shelf optimization software. Although we describe lifting techniques in the context of flux balance analysis, our methods can be used to handle a variety of optimization problems arising from analysis of multiscale network reconstructions.

## Background

Let *S* ∈ **R**^*m*×*n*^ be a stoichiometric matrix that represents a biochemical network consisting of *m* species interacting via *n* reactions. Flux balance analysis (FBA) predicts steady state reaction rates (fluxes) of such a biochemical network by solving the linear program

(1) $$\begin{array}{cc} \underset{v}{\text{maximize}} & \;c^{T}v\\ \text{subject to} & \;\text{Sv}=0,\\ & \;v_{l}\leq v\leq v_{u}, \end{array}$$

where *v*_*l*_,*v*_*u*_ ∈ **R**^*n*^ are lower and upper bounds on the fluxes and *c* represents a biologically motivated objective function. We refer to [1] for details about FBA.

Recently, Thiele et al. [2] described the first genome-scale integrated reconstruction of *E. coli* metabolism and macromolecular synthesis that represents the function of almost 2000 genes. This Metabolic-Expression model explicitly accounts for the demands of macromolecular synthesis at single nucleotide resolution. To enforce consistency between the state of metabolism and macromolecular synthesis, Thiele et al. introduce *coupling constraints* on certain pairs of fluxes (for example, the fluxes for a metabolic reaction and the reaction responsible for synthesizing the enzyme that catalyzes the metabolic reaction [3]):

(2) $$c_{\min}\leq \frac{v_{1}}{v_{2}}\leq c_{\max},$$

where *c*_min_,*c*_max_ > 0. Each coupling constraint can be formulated as a pair of linear inequality constraints, as described later. We predict the steady state reaction rates of such integrated networks by solving the linear program

(3) $$\begin{array}{cc} \underset{v}{\text{maximize}} & \;c^{T}v\\ \text{subject to} & \;\text{Sv}=0,\\ & \;\text{Cv}\leq d,\\ & \;v_{l}\leq v\leq v_{u}, \end{array}$$

where *Cv* ≤ *d* includes constraints equivalent to (2) for many pairs of fluxes.

Given the inherent multiscale nature of integrated reconstructed networks, the constraint matrices of the FBA linear programs (1) and (3) often contain entries that vary over many orders of magnitude. We say that the problems are *poorly scaled*. Conducting FBA for such networks has been unsatisfactory because even state-of-the-art linear programming solvers can produce inaccurate (or erroneously infeasible) results. In particular, for the *E. coli* Metabolic-Expression model, applying CPLEX [4] and Gurobi [5] to (3) with default settings (scaling enabled) has produced results with large constraint violations.

## Implementation

### Scaling techniques

In the context of the simplex method for linear programming, the constraints (including bounds) form a polytope in *n*-space. The condition of a basis matrix associated with a vertex of the polytope provides a quantitative measure of either the “sharpness” or the “flatness” of the vertex. Poorly scaled constraints tend to create a polytope with very sharp and/or very flat vertices. To alleviate numerical difficulties for problem (1), linear programming systems typically compute row and column scaling matrices *D*_*r*_ ∈ **R**^*m*×*m*^ and *D*_*c*_ ∈ **R**^*n*×*n*^ such that the nonzero entries of the scaled constraint matrix *D*_*r*_*SD*_*c*_ are of order 1. Scaling can improve the condition of many bases, but it may be at the expense of making other bases more ill-conditioned (including the optimal basis). For some problems, such as (3), the scaled constraints $D_{r}SD_{c}\bar{v}=0$ may be satisfied accurately by the scaled solution $\bar{v}$, but when the solution is unscaled, $v=D_{c}\bar{v}$ may violate *Sv* = 0 significantly. We refer to [6] for a comprehensive study of scaling and its effects on the performance of the simplex method.

### Lifting techniques

Lifting techniques are commonly used in optimization to create an efficient representation of a feasible set. By using auxiliary variables to “lift” the feasible set into a higher-dimensional space, they can dramatically reduce the computational expense (e.g., see Albersmeyer and Diehl [7], Gouveira et al. [8]). The canonical application is for efficiently representing the cross-polytope, i.e., the set

$$\left\{x\in R^{n}|\sum_{i=1}^{n}|x_{i}|\leq 1\right\}.$$

To represent this set in *n*-dimensional space requires 2^*n*^ constraints of the form

$$\pm x_{1}\pm \cdots \pm x_{n}\leq 1.$$

By introducing *n* new variables *y*_*i*_, thereby lifting the set into 2*n*-dimensional space, we can represent the cross-polytope using 2*n* + 1 constraints:

$$\begin{array}{ll} -y_{i}\leq x_{i} & \leq y_{i},\;i=1,\ldots ,n,\\ y_{1}+\cdots +y_{n} & \leq 1. \end{array}$$

Here we apply lifting techniques to poorly scaled constraints to make the vertices of the “lifted” polytope more regular. Note that small entries in *S* and *C* do not constitute poor scaling unless all entries in a row or column are small. (There are no such rows and columns in our test data, but in general they would be scaled up to have maximum entry 1.) Our explicit aim is to reduce the magnitude of the *largest* matrix entries so that the reformulated constraints do not need scaling.

#### *Mass balance constraints*

In problem (1), the mass balance constraints *Sv* = 0 often contain poorly scaled reactions such as

(4) A+10000B→C+D,

which may represent the synthesis of a macromolecule in a reconstruction. We can decompose such reactions into sequences of reactions involving dummy metabolites with reasonably scaled coefficients. For example, (4) is equivalent to two reactions involving a dummy metabolite $\hat{B}$:

(5) $$A+100\hat{B}\to C+D,\;100B\to \hat{B}.$$

#### *Coupling constraints*

In problem (3), the constraints *Cx* ≤ *d* include equivalents of the coupling constraints (2). These enforce consistency between the states of the metabolic and macromolecular synthesis reactions and are often poorly scaled because reaction rates can vary over many orders of magnitude. For example, two fluxes could be related by

(6) $$0.0001\leq \frac{v_{1}}{v_{2}}\leq 10000.$$

As before, we can decompose these constraints into sequences of constraints involving auxiliary variables with reasonable coefficients. If the second inequality in (6) were presented to our implementation as *v*_1_ ≤ 10000*v*_2_, we would transform it to two constraints involving an auxiliary variable *s*_1_:

(7) $$\begin{array}{l} v_{1}\leq 100s_{1},\;s_{1}\leq 100v_{2}. \end{array}$$

If the first inequality in (6) were presented as *v*_1_ ≥ 0.0001*v*_2_, we would leave it alone, but the equivalent inequality 10000*v*_1_ ≥ *v*_2_ would be transformed to

$$v_{2}\leq 100s_{2},\;s_{2}\leq 100v_{1}.$$

#### *Hierarchical lifting*

Our implementation of lifting techniques uses a parameter *τ*, set to 1024 in our experiments. Constraints containing entries larger than *τ* are reformulated.

Very large entries might require more than one auxiliary variable and constraint. In these cases, we choose the reformulated constraint coefficients to be equally spaced in logarithmic scale. For example, the poorly scaled reaction

$$A+10^{9}B\to C+D$$

(with |10^9^| > *τ*) would be reformulated as

$$\begin{array}{ll} A+1000B_{1} & \to C+D,\;\\ 1000B_{2} & \to B_{1},\;\\ \;1000B & \to B_{2} \end{array}$$

(with |1000|≤*τ*).

#### *Comment*

**Figure 1:**
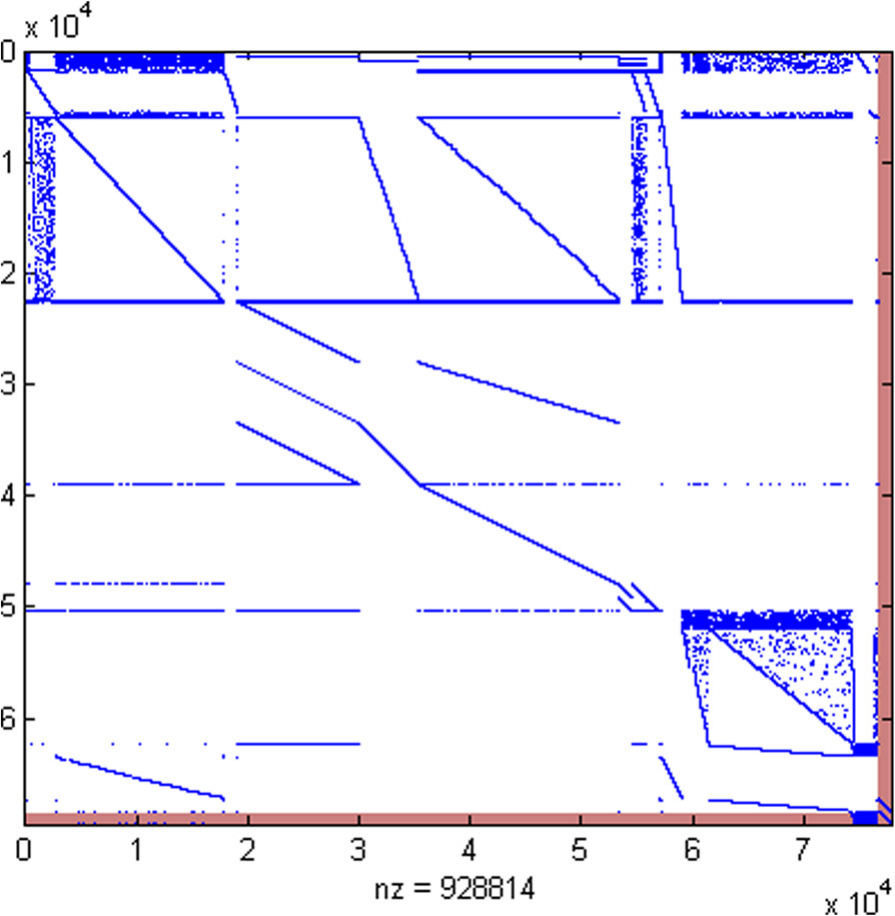
***E. coli *Metabolic-Expression matrix before and after lifting.** Spy plot of the *E. coli* Metabolic-Expression matrix before and after lifting. The red areas were added by the lifting procedure and are very sparse.

Unlike traditional scaling, the above lifting techniques transform poorly scaled constraints without affecting other constraints. The linear program does become larger (more constraints and variables), but the added constraints are extremely sparse and should have little impact on the performance of a typical large-scale solver (see Figure 1). Indeed, the time per iteration for the simplex method could well decrease because smaller “large” entries in the basis matrices typically lead to sparser basis factorizations.

### Iterative refinement

After a simplex solver has returned an allegedly optimal basic solution, the accuracy of satisfying the general linear constraints (*Sv*=0 and *Cv*≤*d* in (3)) could be improved by applying a single step of classical iterative refinement [9], especially if extended precision were available. However, the refined basic solution could well lie outside its bounds, and further simplex iterations would be necessary. Ideally this difficulty would be handled by the simplex solver itself.

We note that more elaborate forms of iterative refinement have been used to improve the accuracy of linear programming solutions. Gleixner et al. [10] describe an incremental precision-boosting procedure that solves a sequence of linear programs, each attempting to correct the error in the previous optimal solution. The Zoom procedure of Saunders and Tenenblat [11] is an analogous strategy for interior methods.

### Implementation in the openCOBRA toolbox

Lifting techniques for poorly scaled reactions and coupling constraints have been implemented in the openCOBRA toolbox 2.05 [12], a Matlab package for constraint-based reconstruction and analysis of biochemical networks. Algorithm 1 summarizes the main steps. Our implementation makes efficient use of auxiliary variables by reusing them if possible. Suppose metabolite *A* participates in more than one reaction with large stoichiometric coefficients. We can use the same auxiliary variable to decompose all reactions involving metabolite *A*, thereby keeping problem size to a minimum.

To benefit from solving the reformulated problem, we must disable scaling and any “presolve” option that would permit reaggregation of constraints. Our implementation automatically sets these options for CPLEX and Gurobi. 

## Results and discussion

We use our implementation of lifting techniques to conduct FBA on two Metabolic-Expression models of *E. coli*[2]. The models (ME76664 and ME76589) represent the function of almost 2000 *E. coli* genes and involve 62212 metabolites, with 6087 coupling constraints *Cv* ≤ *d* to enforce consistency between the predicted steady states of both metabolism and macromolecular synthesis. The first model (ME76664) accounts for 76664 reactions, and the second (ME76589) accounts for 76589 reactions. Because of the dependencies between pairs of metabolic reactions and macromolecular synthesis reactions, the resulting flux balanced steady state *v* has reaction rates that vary by four orders of magnitude [2]. Both models have about 41,000 large matrix entries (exceeding *τ* = 1024), with 1825 entries exceeding 10^5^ and biggest entry 8×10^5^.

Conducting FBA on ME76664 using the CPLEX and Gurobi simplex and barrier solvers with default settings (including scaling) resulted in erroneous reports of infeasibility or “optimal” solutions that were significantly infeasible. Our own simplex solver SQOPT [13] with scaling activated would solve the scaled problem well, but unscaling would magnify the infeasibilities.

With the CPLEX solvers, our lifting techniques eliminate infeasible reports and significantly reduce the infeasibility of the computed steady states; see Table 1 and Table 2. Note that most of the “barrier iterations” are really simplex iterations required by *crossover* (the procedure for finding a basic solution from the barrier solution). These do not alter the optimal objective value and may not be essential in practice.

**Table 1:** FBA results for ME76664 before and after lifting

**68299 rows**	**Simplex**		**Barrier**	
**76664 columns**	**Before**	**After**	**Before**	**After**
Iterations	48603	58288	56490	9985
CPU time	242	292	384	93
Infeasibilities	1.3×10^−4^	2.9×10^−6^	1.4×10^−1^	3.4×10^−6^

FBA results for the *E. coli* Metabolic-Expression model ME76664 using CPLEX primal simplex and barrier solvers. Iterations, time, and sum of infeasibilities before and after lifting. The iterations in columns 4 and 5 include about 100 for the barrier solver and the remainder for the simplex crossover.

**Table 2:** FBA results for ME76589 before and after lifting

**68299 rows**	**Simplex**		**Barrier**	
**76589 columns**	**Before**	**After**	**Before**	**After**
Iterations	22649	70786	22816	32278
CPU time	209	601	350	584
Infeasibilities	9.7×10^−4^	9.8×10^−7^	7.1×10^−2^	6.2×10^−5^

FBA results for the *E. coli* Metabolic-Expression model ME76589 using CPLEX primal simplex and barrier solvers. Again, most of the barrier iterations are for the simplex crossover.

**Figure 2:**
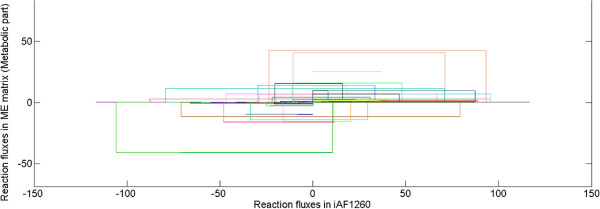
**Flux variability analysis of the *E. coli *Metabolic-Expression model.** Minimum and maximum flux for iAF1260 (which only accounts for metabolic reactions) versus the minimum and maximum flux for the Metabolic-Expression model. Each colored box corresponds to a different reaction in metabolism. The boxes are always longer on the axis for the metabolic model (iAF1260) than on the axis for the Metabolic-Expression model. This demonstrates that increasing the comprehensiveness of the model toward whole cell modeling leads to a substantial shrinkage of the steady state solution space. (Fluxes are plotted in $\text{mmol}\cdot g_{\text{dw}}^{-1}\cdot {\text{hr}}^{-1}$).

We also used lifting to conduct flux variability analysis (FVA) [14] for the ME76664 model and obtained biologically consistent results (see Figure 2). We compared the flux span of each metabolic reaction in ME76664 with the flux span of the corresponding reaction in the *E. coli* metabolic model (iAF1260) [15]. The chief difference between these two models is that in ME76664 the metabolic building blocks (e.g., amino acids) are used to synthesize the metabolic enzymes, which in turn catalyze the metabolic reactions, while in iAF1260 the building blocks are collected in a static biomass reaction. Artifacts with FBA on metabolic models, such as thermodynamically infeasible flux around stoichiometrically balanced reaction cycles, are eliminated for all enzyme-catalyzed reactions in ME76664, as the coupling constraints penalize high flux rates. These constraints also restrict the maximum possible flux rates through enzyme catalyzed reactions due to the demand-supply challenge for the building blocks, thus limiting the set of possible transcriptomes and proteomes of the model. Overall, the feasible steady state solution space is substantially reduced in ME76664 compared to the metabolic model alone.

Tables 3 and 4 summarize 15 FVA runs using the CPLEX simplex and barrier solvers. For the simplex method (Table 3) we see that lifting reduces the infeasibilities of the computed steady states and also stabilizes the number of simplex iterations. For the barrier method (Table 4) the effects of lifting are much more varied. The feasibility of the computed steady state is sometimes improved but the lifted problem can take much longer to solve. Evidently the CPLEX barrier solver (with crossover) does not perform reliably on ME76664 with or without lifting.

**Table 3:** FVA results (simplex solvers) for ME76664 before and after lifting

		**Iterations**		**Infeasibilities**
**Flux**	**Before**	**After**	**Before**	**After**
1	22510	44496	1.1×10^−4^	7.6×10^−5^
5001	30405	40318	1.5×10^−4^	9.6×10^−5^
10001	34963	41231	9.4×10^−2^	8.2×10^−5^
15001	103210	41891	4.5×10^−5^	9.4×10^−6^
20001	120089	40587	8.8×10^−2^	8.3×10^−5^
25001	30786	41161	1.7×10^−4^	8.3×10^−5^
30001	55177	40534	9.8×10^−2^	8.1×10^−5^
35001	68760	40933	1.3×10^−4^	8.3×10^−5^
40001	30360	40778	1.2×10^−4^	1.6×10^−5^
45001	107485	40905	3.1×10^−5^	8.3×10^−5^
50001	32553	40360	9.7×10^−2^	8.5×10^−5^
55001	20661	39909	9.5×10^−5^	5.7×10^−5^
60001	25477	39830	9.4×10^−2^	8.6×10^−5^
65001	139251	42230	2.9×10^−5^	8.4×10^−5^
70001	137611	42389	6.2×10^−5^	8.3×10^−5^
75001	40930	41139	4.0×10^−5^	8.2×10^−5^

FVA results for the *E. coli* Metabolic-Expression model ME76664 using the CPLEX simplex solvers. Iterations and sum of infeasibilities for dual simplex (default) before lifting and for primal simplex after lifting. The first column lists which variable is being maximized. Lifting helps the CPLEX simplex solvers.

**Table 4:** FVA results (barrier solver) for ME76664 before and after lifting

		**Iterations**		**Infeasibilities**
**Flux**	**Before**	**After**	**Before**	**After**
1	76669	23084	9.4×10^−2^	9.0×10^−2^
5001	34721	66731	4.7×10^0^	8.8×10^−2^
10001	58672	85819	9.3×10^−2^	9.3×10^−4^
15001	28032	47901	9.7×10^−2^	1.6×10^−2^
20001	18715	30433	3.2×10^−2^	3.0×10^−2^
25001	12224	17973	3.5×10^2^	8.8×10^−2^
30001	19621	35111	8.8×10^−2^	3.1×10^−2^
35001	6743	7630	1.0×10^−2^	6.8×10^−2^
40001	47609	4111	8.8×10^−2^	9.4×10^−2^
45001	9117	9980	1.0×10^−1^	3.2×10^−2^
50001	9567	49350	9.5×10^−2^	9.5×10^−2^
55001	23985	13362	9.6×10^−2^	1.1×10^−1^
60001	99067	27075	1.1×10^−1^	9.1×10^−2^
65001	44796	11509	3.1×10^−1^	8.9×10^−2^
70001	17045	14393	4.0×10^−2^	6.5×10^−2^
75001	20790	14908	9.0×10^−2^	5.6×10^−6^

FVA results for the *E. coli* Metabolic-Expression model ME76664 using the CPLEX barrier solver. Iterations and sum of infeasibilities before and after lifting. The first column lists which variable is being maximized. Columns 2 and 3 list the total iterations for the barrier solver and the simplex crossover (with barrier requiring only about 100 iterations in all cases). CPLEX barrier appears less reliable than the CPLEX simplex solvers on this model.

## Conclusions

We described techniques that enable off-the-shelf optimization software to be applied to multiscale network reconstructions, such as integrated networks that represent both metabolism and macromolecular synthesis. The techniques enable accurate FBA and FVA of an integrated model of metabolism and macromolecular synthesis in *E. coli*, previously impossible because of numerical difficulties encountered by solvers.

As *in silico* biologists create increasingly complex models that capture more of the multiscale nature of biological systems [16], the optimization problems that arise during the analysis of these models will also become increasingly poorly scaled. We are aware of researchers resorting to specialized packages such as [17] that rely upon rational arithmetic to obtain exact solutions to the FBA and FVA linear programs. Such solvers are likely to be prohibitively slow for analyzing larger, more comprehensive reconstructed networks. A more practical approach is to employ quadruple-precision arithmetic, which is increasingly available in Fortran and C compilers and is valuable even when implemented in software. In the meantime, our techniques enable the constraint-based modeling community to analyze increasingly sophisticated and comprehensive models of biological systems with improved efficiency and reliability. They could also be combined with the refinement approach of Gleixner et al. [10].

## Availability and requirements

Lifting techniques for poorly scaled reactions and coupling constraints have been implemented in the openCOBRA toolbox 2.05 [12], a MATLAB package for constraint-based reconstruction and analysis of biochemical networks.

**Project name:** openCOBRA toolbox

**Project home page:**http://opencobra.sourceforge.net/

**Operating system:** platform independent

**Programming language:**MATLAB

**Other requirements:**MATLAB 2008a or higher

**License:** GNU GPLv3

**Any restrictions to use by non-academics:** A separate license must be acquired.

## Competing interests

The authors declare that they have no competing interests.

## Authors’ contributions

YS developed and implemented the lifting techniques. YS and MAS wrote the manuscript. RMTF and IT provided examples, interpreted results, and edited the manuscript. All authors read and approved the final manuscript.
